# Lévy Walk Navigation in Complex Networks: A Distinct Relation between Optimal Transport Exponent and Network Dimension

**DOI:** 10.1038/srep17309

**Published:** 2015-11-25

**Authors:** Tongfeng Weng, Michael Small, Jie Zhang, Pan Hui

**Affiliations:** 1 HKUST-DT System and Media Laboratory, Hong Kong University of Science and Technology, HongKong; 2 The University of Western Australia, Crawley, WA 6009, Australia; 3 Centre for Computational Systems Biology, Fudan University, China

## Abstract

We investigate, for the first time, navigation on networks with a Lévy walk strategy such that the step probability scales as *p*_*ij*_ ~ *d*_*ij*_^–*α*^, where *d*_*ij*_ is the Manhattan distance between nodes *i* and *j*, and *α* is the transport exponent. We find that the optimal transport exponent *α*^opt^ of such a diffusion process is determined by the fractal dimension *d*_*f*_ of the underlying network. Specially, we theoretically derive the relation *α*^opt^ = *d*_*f*_ + 2 for synthetic networks and we demonstrate that this holds for a number of real-world networks. Interestingly, the relationship we derive is different from previous results for Kleinberg navigation without or with a cost constraint, where the optimal conditions are *α* = *d*_*f*_ and *α* = *d*_*f*_ + 1, respectively. Our results uncover another general mechanism for how network dimension can precisely govern the efficient diffusion behavior on diverse networks.

Networks are ubiquitous in a vast range of natural and man-made systems ranging from the Internet through human society to the oil-water flow^1^,^2^,^3^,^4^. Since the discovery of the scale-free property^5^ and the small-world phenomenon^6^, network science has fundamentally altered our view of diverse real-world systems, which provides an abundance of statistics to characterize and interpret the relations encoded in their network representations. Recently, intensive attention has been dedicated to dynamical processes taking place on networks beyond purely topological aspects^7^,^8^,^9^,^10^,^11^,^12^,^13^. In particular, it is of great interest to investigate navigation in routing and delivery of information efficiently on social, biological and technological networks^12^,^13^.

For navigability of networks, Roberson *et al.* claims that when only local information is available, the optimal condition is the addition of long-range links taken from the distribution 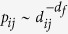, where *d*_*f*_ is the fractal dimension of the underlying network^8^. Later, Kosmidis *et al.* find that the optimal condition is 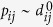 based on the global information of the network structure^9^. Recently, unlike the previous unconstrained situation, Li *et al.* provide the design principles for optimal transport networks under imposition of a cost constraint of long-range links, where the best condition is obtained with the long-range links taken from 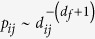, regardless of the strategy used based on local or global information of the whole network^10^,^11^. In fact, all these strategies have some common characteristics that the efficient mobility is achieved by choosing one of the available links of a site to follow (based on local or global knowledge of the network structure) that potentially optimizes the path. Very recently, the navigation strategy of a Lévy walk has been introduced on networks for which the transition probability follows a power law function of distance, i.e., 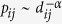, where *α* is the transport exponent^14^. In contrast to the previous strategies that require optimizing the path at each step, a Lévy walk performs jumps on networks randomly. Various studies have demonstrated that *α* ≈ 2 is the optimal value for animals and human foraging under general circumstances^15^,^16^,^17^,^18^. However, the exact interplay between network structure and the optional transport exponent of a Lévy walk is still missing.

In this paper, we investigate the Lévy diffusion processes on networks and find that the optimal exponent of such diffusion process occurs at *α* = *d*_*f*_ + 2, where *d*_*f*_ is the fractal dimension of the underlying network, in contrast to the previous findings, where *α* = *d*_*f*_^7^,^8^ and *α* = *d*_*f*_ + 1^10^,^11^, respectively. We explore the origin of such behavior using the extensional concept of entropy rate incorporating the cost of long range jumps and show that it is an universal principle widely existing on a variety of physical networks ranging from social, technological to biological networks. Our results help unravel another general mechanism of exactly how network dimension governs efficient diffusion processes. Furthermore, our results indicate that this efficient global approach of mobility only depends on the dimension of the underlying network, sometimes that is impossible to obtain merely based on limited and local information.

## Results

### Diffusion process of Lévy walks

We start from a network consisting of *N* nodes. The network is fully described by a symmetric adjacency matrix *A* with elements *a*_*ij*_ = 1 if nodes *i* and *j* are connected and *a*_*ij*_ = 0 otherwise. The diffusion processes that we study is a Lévy walk on this network exerting a power-law transition probability with the distance given by^14^




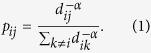




**Figure 1:**
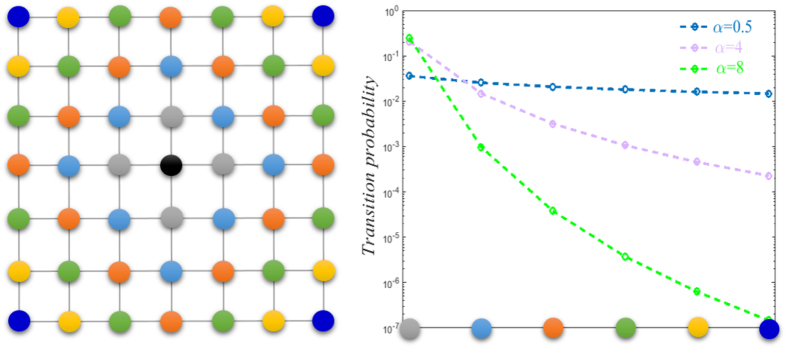
Modelling transition probability of a Lévy walk versus shortest path length at the seeding node. (Left panel) A 2D lattice composed of 49 nodes and 84 edges. Distinct colored nodes represent the different shortest path lengths from the seeding node (i.e., the central node). (Right panel) The transition probability decays with respect to the shortest path length under small, medium, large transport exponents *α*, respectively.

In this context, the walker usually has a larger transition probability to nearest neighbors, whereas the transition probability tends to be smaller for indirectly linked nodes. The tradeoff between short-range and long-range distances of hopping in one step is fully controlled by the transport exponent *α* that varies in the interval 0 ≤ *α* < ∞. Figure 1 illustrates the transition probabilities versus the shortest path lengths with respect to the transport exponent *α*. Specially, with a small *α*, the walker can visit the nearest neighbors and neighbors that are far away with approximately equivalent probability. By contrast, the walker possibly only jumps to the nearest neighbors at an extremely large *α*, which corresponds to the generic random walk^19^. Such mobility behavior is comparable to that of Lévy flights widely reported in the literature, for instance, foraging by animals^16^ and human^17^, and even the migration of effector T cells^18^, which is an efficient navigation strategy in searching and foraging under general circumstances.

**Figure 2:**
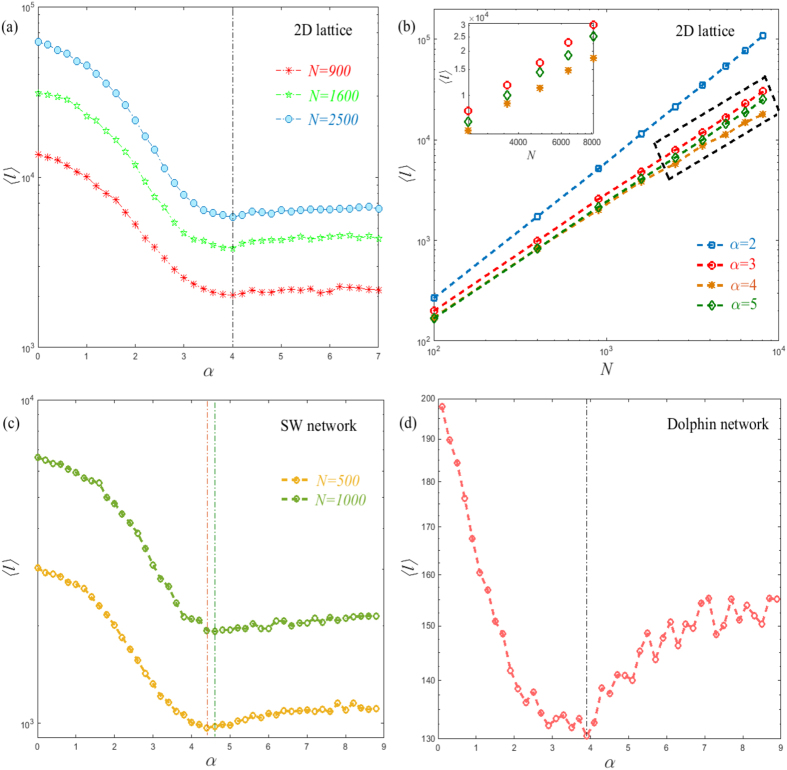
The expected delivery distance 〈*l*〉 as a function of *α* for the Lévy walk on: (a) a 2D lattice, (c) small-world network with rewiring probability *p* = 0.08^6^, and (d) a social network: frequent associations between 62 dolphins in a community living in Doubtful Sound^20^. To implement the information propagation, the source and target nodes are selected randomly. The position of the minimum delivery distance 〈*l*〉 is marked by the dotted line. In (**b**), we show the expected delivery distance 〈*l*〉 as a function of 2D lattice size *N* for different *α*. The profile of *α* = 4 increases slower with *N* compared to any other value of *α*. Inset: higher magnification view of the boxed area. To obtain these results, each data point is the average of 5,000 runs.

Clearly, the transport exponent *α* plays a fundamental role in shaping the behavior of the Lévy walk. In order to explore how the critical behavior of a Lévy walk changes with respect to the transport exponent *α*, we first address such mobility on two synthetic networks (i.e., 2D lattices^10^ and the small-world network^6^) and one social network (i.e., frequent associations between 62 dolphins in a community living in Doubtful Sound^20^). We use the expected delivery distance 〈*l*〉 to characterize the efficiency of a Lévy walk and perform extensive simulations on each of them. The expected delivery distance 〈*l*〉 represents the number of paths required, on average, to deliver the message from a source to target chosen randomly on the network. The result presented in Fig. 2(a) clearly indicates the presence of a minimum 〈*l*〉 for different lattice sizes *N* at the same exponent *α* = 4, whereas the delivery distance 〈*l*〉 is significantly larger, when *α* ≠ 4. We also notice that, when *α* is extreme large, the Lévy walk degenerates to the generic random walk. So, the delivery distance 〈*l*〉 approaches a fixed value for *α* > 5, see in Fig. 2(a), as expected. Moreover, we test behaviors of 〈*l*〉 as the function of network size *N* for different values of *α*, see in Fig. 2(b). Our results show that, when *α *≠ 4, the expected delivery distance 〈*l*〉 follows a power law with network size *N*. In contrast, the profile of 〈*l*〉 vs *N* exhibits a less rapid than a power law behavior for *α* = 4. This provides further support that the optional exponent of a Lévy walk on 2D lattices occurs at the position of *α* = 4. Meanwhile, similar behaviors are also displayed by the small-world network and the dolphin network, see in Fig. 2(c,d). Their profiles show the existence of a clear minimum in the average delivery distance. Interestingly, positions of their minimum *α*^opt^ appear very distinct. In particular, for the small-world network of size *N* = 500, *α*^opt^ approximately equals 4.4, while *α*^opt^ is 3.9 for the dolphin network.

### Entropy rate of Lévy walks

To investigate these phenomena theoretically, we adopt the concept of entropy rate to characterize the efficiency of Lévy walk on a network. The entropy rate measures the minimal amount of information necessary to describe the diffusion process^21^,^22^. In this context, a higher entropy rate represents an efficient spreading of the diffusion process over the network^21^,^22^,^23^,^24^. For a given diffusion process with the transition probability {*p*_*ij*_}, its entropy rate is defined as follows:




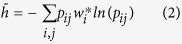




where 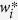 is the *i*th component of the stationary distribution. Unfortunately, such a definition of entropy rate may suffer from some limitations when applied directly to diffusion processes having long-range hopping such as the Lévy walk^14^ and the PageRank Algorithm^25^. Under this definition, the maximal entropy rate of the Lévy walk will occur at *α* = 0, which is trivial as the definition does not take into account the cost of long-range hopping^26^. To overcome such a drawback, we provide a modified definition of entropy rate as follows:




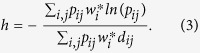




**Figure 3:**
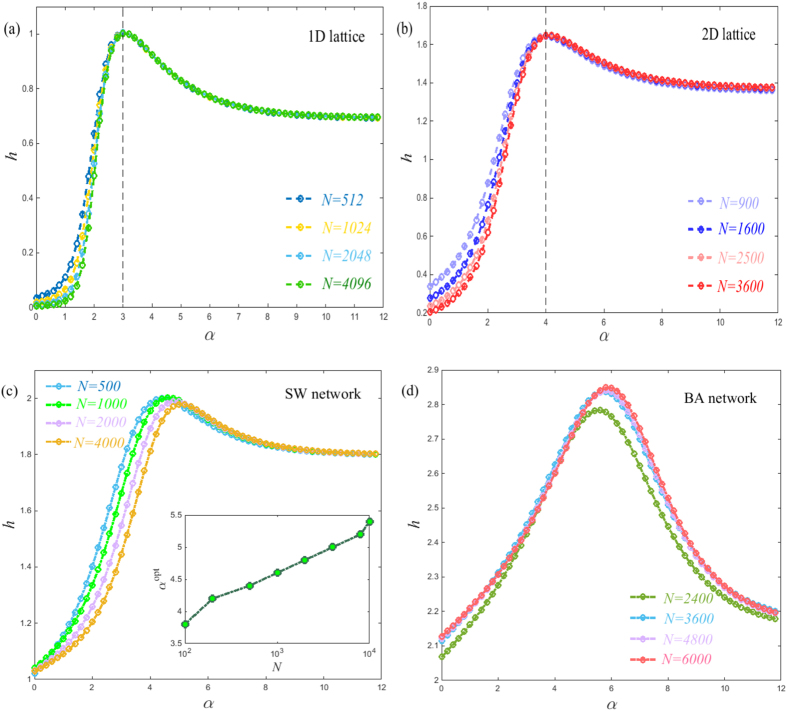
Top: the entropy rate *h* as a function of *α* for (a) one dimension lattice (*d* = 1) and (b) two dimension lattice (*d* = 2) with different sizes *N*. The maximal entropy rate is observed at *α*^opt^ = *d* + 2 for them, marked by the dotted lines. Bottom: the entropy rate *h* in two synthetic networks: the BA network generated with the preferential attachment method^5^ and the SW network with the rewiring probability *p* = 0.08^6^. The inset shows the position of the maximum *α*^opt^ as a function of the network size *N*.

The sum in the denominator quantifies the cost of long-range hopping for the Lévy walk. Specifically, when *α* = 0, we obtain *h* = *ln*(*N* − 1)/〈*d*〉, where 〈*d*〉 is the average shortest path length on the whole network. Therefore, it will be around 1 for small-world networks, as 〈*d*〉 ≈ *ln*(*N*). In contrast, when *α* → ∞, the transition probability of the Lévy walk, Eq. (1), degenerates to *p*_*ij*_ = *a*_*ij*_/*k*_*i*_, where *k*_*i*_ is the degree of node *i*. In this situation, it is easy to verify that the modified definition of entropy rate, Eq. (3), is equivalent to the generic entropy rate, Eq. (2), as expected. Figure 3 shows our modified entropy rate *h* with respect to the transport exponent *α* on lattice models. Interestingly, it is shown that the entropy rate exhibits a single maximum at *α*^opt^ = *d* + 2 on lattice models, which implies that the optimal diffusion process of a Lévy walk heavily depends on the dimension of the lattice model. Meanwhile, the entropy rate approaches a fixed value, (i.e., the entropy rate of a random walk) when *α* is higher than 8, which is consistent with our previous argument. Moreover, exactly the same behavior is displayed by two other synthetic networks, the Barabási-Albert (BA) model^5^ and the previous small-world (SW) network^6^, see in Fig. 3(c,d). In all cases examined, *h* appears to be a convex smooth function of *α* with a clear maximum. The location of the maximum also depends on the dimension of the underlying network. In particular, the optional value *α*^opt^ progressively increases as the size *N* of the small-world network increases. It hints that the larger the size *N*, the larger the fractal dimension *d*_*f*_ of the small-world network is, which is consistent with the result as suggested in^27^. Consequently, at this point we conjecture that the relation *α*^opt^ = *d*_*f*_ + 2 will be universal across a variety of networks with the fractal dimension *d*_*f*_. For calculating the fractal dimension of a network, the classical approach is based on the box-counting method given by^28^:




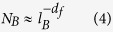




where *N*_*B*_ is the minimum number of boxes needed for covering the entire network with the box size *l*_*B*_. For achieving the minimal number *N*_*B*_, several other approaches have been reported^20^,^29^,^30^.

### The relation between the optimal transport exponent and network dimension

In the following, we present analytical arguments to demonstrate our conjecture that, the optimal exponent *α*^opt^ of Lévy walk occurs at *α*^opt^ = *d*_*f*_ + 2, *d*_*f*_ being the dimension of the underlying network. Assuming that the fractal network is finite consisting of *N* nodes and the stationary distribution of Lévy walk on each node *i* is equiprobable (i.e., 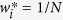). The network diameter *L* can be approximated as 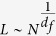. Then, the entropy rate of a Lévy walk (see methods) becomes




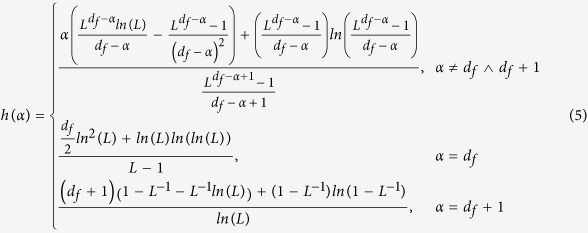




It is easy to prove that entropy rate *h* is a continuous function of the transport exponent *α* such that 
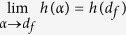
 and 
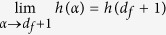
 hold, respectively. We thus obtain the maximal entropy rate of a Lévy walk at the position of (see methods)




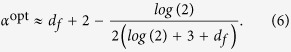




The preceding equation indicates that the optimal exponent occurs at the position of *α*^opt^ ≈ *d*_*f*_ + 2, which further verifies our previous numerical simulations (see Fig. 3). It is very interesting to note that the optimal exponent of a Lévy walk only depends on the fractal dimension rather than other statistics of the network structure. This may, to some extent, explain why the Lévy walk is a global navigation strategy, which has a dramatic difference from the widely discussed random walk whose maximal entropy rate heavily relies on the degree-degree correlations of network structure as suggested in^22^.

**Figure 4:**
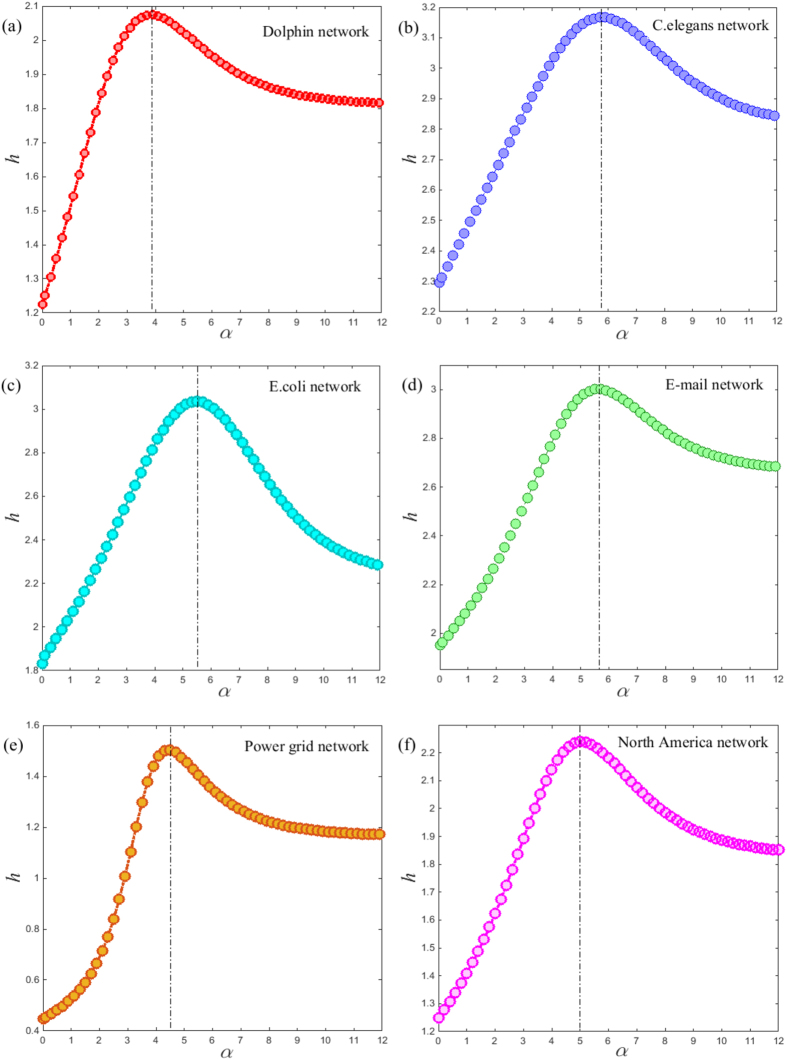
Entropy rate *h* as a function of *α* for six real networks with the well defined fractal dimension. The maximum of entropy rate occurs at position of *α*^opt^ = 3.9 (Dolphin), 5.7 (C. elegans), 5.5 (E. coli), 5.6 (E-mail), 4.5 (Power grid) and 5 (North America), respectively, marked by the solid lines. Their fractal dimensions are *d*_*f*_ = 1.88 (Dolphin), 3.7 (C. elegans), 3.45 (E. coli), 3.69 (E-mail), 2.42 (Power grid) and 3 (North America), with reference to^20^,^28^,^29^,^30^,^35^.

Finally, we consider the application to several real networks including social (e-mail^31^ and dolphin^20^), biological (C. elegans^32^, and E. coli^29^) and technological networks (power grid^33^ and North America^34^) to further demonstrate the relation between network dimension and the optimal exponent of a Lévy walk. All these real networks have a well defined fractal dimension^20^,^28^,^29^,^30^,^35^. We calculate the entropy rate of the Lévy walk on these real networks based on Eq. (3). It is shown that their entropy rate exhibits a similar profile that markedly increases on small exponents and then smoothly decreases to the fixed value. As expected, the emergence of the maximal entropy rate has some connection with the fractal dimension of the underlying networks, (see Fig. 4). More precisely, we find that the relation *α*^opt^ = *d*_*f*_ + 2 is approximately established across all these real networks, which further supports our previous findings. Results suggest that, the scaling property of the transition probability, (i.e., 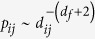), is the most optimal way to obtain mobility on diverse real networks while ensuring efficient information spreading. Moreover, we note that when *α* = 0, the entropy rate of these real networks is higher than 1 with the exception of the Power grid network, see Fig. 4. This is because the average shortest path length 〈*d*〉 of the Power grid network is 19, and this network has no small-world characteristics. In this sense, the profile of entropy rate further shows whether or not the underlying network has the small-world feature.

## Discussion

In summary, we have studied navigation of diffusion processes on networks with long-range transition taken from a power-law distribution. We find that the best transportation condition is obtained with an exponent *α* = *d*_*f*_ + 2, where *d*_*f*_ is the fractal dimension of the underlying network. We use the entropy rate to investigate the origin of such scaling phenomenon and we show that such relation holds for a variety of real networks. Our finding is different from the results obtained for Kleinberg navigation and for the constraint of long-range connections, where the optimal conditions are *α* = *d*_*f*_^8^ and *α* = *d*_*f*_ + 1^11^, respectively. Our results offer a useful framework to construct an efficient way of mobility on social, biological and technological networks, further enriching our understanding of interplay between dynamics and structure. Moreover, our modified definition of entropy rate can provide an effective paradigm to characterize diffusion processes on networks having long-range jumps, such as the PageRank Algorithm^25^.

## Methods

### The analytic expression of entropy rate of a Lévy walk

Assuming that the fractal network is finite and consists of *N* nodes and that the stationary distribution of the Lévy walk on each node *i* is equiprobable (i.e., 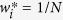). The network diameter *L* can be approximated as 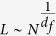. Then, the modified entropy rate of a Lévy walk can be rewritten as




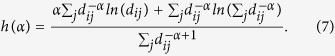




Approximating *L* as a continuous variable, the term 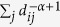 consequently scales as^8^,^10^,^11^,^36^




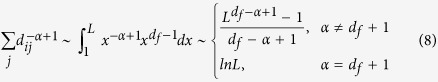




Repeating a similar calculation for the terms 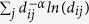 and 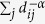, we obtain




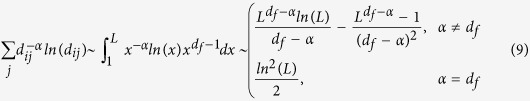







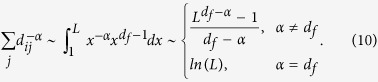




Substituting them together with expression (8) into Eq. (7), the entropy rate of a Lévy walk becomes




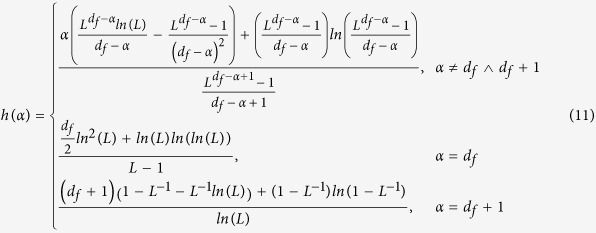




### The optional exponent of a Lévy walk on networks

We simplify Eq. (5) with a few simple algebraic manipulations. For *α* < *d*_*f*_ + 1, the entropy rate tends to 0, when *L* → ∞. Conversely, for *α* ≥ *d*_*f*_ + 1, empirically we find that the simulation values of the term 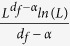 is far less than that of the term 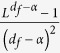 and can be neglected in the analysis. In this context, when *L* → ∞, Eq. (5) reduces to
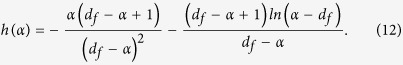


Then, it is possible to obtain the derivative of *h*(*α*)




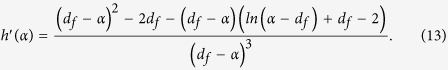




Using the second-order Taylor expansion of the term *ln*(*α* − *d*_*f*_), we thus obtain the maximal entropy rate of a Lévy walk at the position of




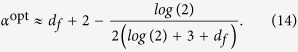




## Additional Information

**How to cite this article**: Weng, T. *et al.* Lévy Walk Navigation in Complex Networks: A Distinct Relation between Optimal Transport Exponent and Network Dimension. *Sci. Rep.* **5**, 17309; doi: 10.1038/srep17309 (2015).
